# Dehydroepiandrosterone (DHEA) replacement decreases insulin resistance and lowers inflammatory cytokines in aging humans

**DOI:** 10.18632/aging.100327

**Published:** 2011-05-10

**Authors:** Edward P. Weiss, Dennis T. Villareal, Luigi Fontana, Dong-Ho Han, John O. Holloszy

**Affiliations:** ^1^ Division of Geriatrics and Nutritional Sciences, Department of Medicine, Washington University School of Medicine, St. Louis, MO 63110, USA;; ^2^ Department of Nutritional and Dietetics, St. Louis University, St. Louis, MO 63104, USA;; ^3^ Geriatrics, University of New Mexico School of Medicine and New Mexico VA Health Care System, NM 87108, USA;; ^4^ Division of Nutrition and Aging, Instituto Superiore di Sanità Rome Italy 00161

**Keywords:** aging, insulin resistance, interleukin 6, tumor necrosis factor α

## Abstract

Plasma dehydroepiandrosterone (DHEA) decreases ~80% between ages 25 and 75 yr. In a preliminary study, we found that 6 mo of DHEA replacement improved insulin action in elderly individuals. The purpose of the present larger, randomized double-blind study was to determine whether a longer period of DHEA replacement improves glucose tolerance. Fifty-seven men and 68 women aged 65 to 75 yr were randomly assigned to 50 mg DHEA or placebo once daily. Year one was a randomized, double blind trial. Year 2 was an open label continuation. DHEA replacement improved glucose tolerance in participants who had abnormal GT initially, reduced plasma triglycerides, and the inflammatory cytokines IL6 and TNFα.

This trial was registered at clinicaltrials.gov as NCT00182975.

## INTRODUCTION

Dehydroepiandrosterone (DHEA) and its sulfated form (DHEAS), which will be referred to together as DHEA, are secreted by the zona reticularis of the adrenal cortex only in humans and related primate species [1]. DHEA is present in far higher concentrations in plasma than any other steroid hormone [1]. Adrenal production of DHEA begins during puberty, peaks at ~20 yr and begins to decline with aging beginning at ~25 yr [2,3]. This decline is progressive and severe, resulting in a plasma DHEA level by age 75 yr that is ~80% lower than at 20 yr [2]. It has been reported that DHEA level predicts longevity in men [4]. Studies on laboratory rodents have shown that DHEA treatment reduces total and visceral fat accumulation and protects against development of muscle insulin resistance in response to a high fat diet [5,6], with advancing age [7], and in genetic obesity [8,9].

In a preliminary study to test the hypothesis that, as in rodents, DHEA replacement reduces adiposity and improves insulin action, we evaluated the effects of six months of DHEA treatment (50 mg/day) in a randomized controlled trial on 56 elderly women and men [10]. The DHEA treatment induced significant decreases in abdominal fat and in the insulin area under the oral glucose tolerance curve, without a change in glucose levels, providing evidence for an increase in insulin sensitivity. The present study was undertaken to determine whether a longer period of DHEA replacement improves both insulin action and glucose tolerance in elderly women and men.

## RESULTS

### Study Participants

**Figure 1.**
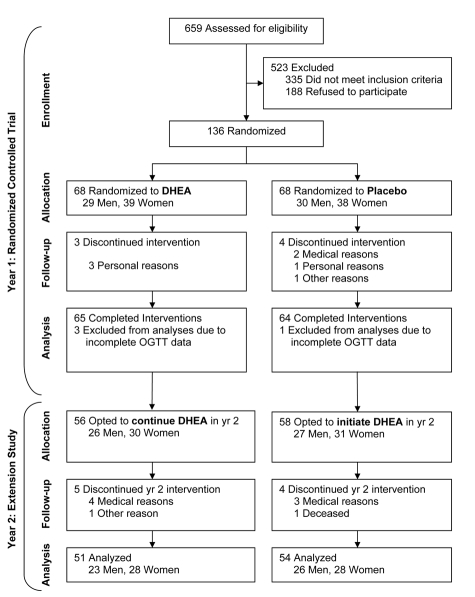
Consort diagram indicating sample sizes at each stage during the study

Of the 659 people who initially expressed interest in the study, 335 did not meet the inclusion criteria and 188 chose not to participate (Figure 1). The remaining 136 eligible volunteers were randomized to one year of DHEA replacement, 50 mg daily, or placebo. After completing the 12 mo randomized study, 112 of the participants volunteered to continue in an open-label study for an additional 12 mo. In the open-label study, the participants who had been randomized to DHEA replacement continued taking DHEA for a second year, while those who had been in the placebo group crossed over to DHEA replacement for 12-mo.

There were no significant differences between the DHEA and placebo groups in gender distribution, age or body mass index (BMI) (Table 1). More of the participants in the placebo group were on anti-dyslipidemic medications (Table 1). Seventy-one percent of the participants in the DHEA group had abnormal oral glucose tolerance tests (2 hr glucose value above 140 mg/dl) initially compared to 52% of those in the placebo group (Table 1).

**Table 1.** Characteristics of the Participants

	DHEA	Placebo	Between Group *P* value
Sex Men Women	29 33	28 35	0.89 0.81
Age, yr	70 ± 3	70 ± 3	0.84
BMI, kg/m^2^	28.4 ± 5.6	27.6 ± 4.9	0.40
Medication use Antidyslipidemic Antihypertensive	19 32	31 30	0.03 0.66
Glucose tolerance Normal glucose tolerance Abnormal glucose tolerance	18 44	30 33	0.08 0.21
History of cardiovascular disease	8	8	0.97
Abbreviations: DHEA, dehydroepiandrosterone; BMI, body mass index

### Complicance

Based on pill count, the percentage of prescribed doses taken averaged 94 ± 0.4% in the DHEA and 96 ± 0.4% in the placebo group.

### Physical Activity and Energy Intake

Energy intake, estimated from food records, averaged 2191 ± 62 kcal/day before and 2181 ± 67 kcal/day after 12 mo of DHEA replacement, and 2116 ± 56 kcal/day before and 2151 ± 68 kcal/day after 12 mo in the placebo group.

### Body Weight and Composition

The men in the DHEA group had a small but significant decrease in body weight during the first year of DHEA replacement, while the men in the placebo group gained weight (Table 2). The DHEA replacement resulted in small but significant decreases in body fat percentage and total fat mass in the male participants, while the men in the placebo group had small but significant increases (Table 2). Trunk and appendicular fat masses showed a similar reduction (Table 2). Abdominal visceral fat, evaluated using MRI, underwent a very small, but statistically significant, decrease in the men in the DHEA group; however, the difference between the changes in visceral fat between the DHEA and placebo groups was not significant (Table 2). There were no significant changes in body weight in the women in either the DHEA or placebo group. The only significant change in body composition in the women was a small increase in fat free mass in the DHEA group.

**Table 2.** Body Weight and Body Composition Before and After 12 months of DHEA

		Men						Women				
	Group		Baseline	12 Mos	Between-group				Baseline	12 Mos	Between-group	
					Difference	P					Difference	P
Body weight, kg	DHEA Placebo		88.2±2.5 83.8±2.2	87.3±2.6* 84.5±2.2	-1.6±0.6	0.008			72.5±2.9 75.1±3.0	72.9±2.9 75.0±3.0	0.5±0.7	0.47
Body fat percentage	DHEA Placebo		28.0±1.0 25.3±1.2	27.2±1.1* 25.9±1.2*	-1.3±0.4	0.001			39.1±1.1 40.0±1.1	38.7±1.1 40.3±1.2	-0.7±0.4	0.09
Fat mass, kg	DHEA Placebo		25.2±1.5 21.7±1.5	24.4±1.6* 22.4±1.5*	-1.6±0.5	0.001			29.1±1.9 30.9±2.0	29.0±1.9 31.1±2.0	-0.3±0.5	0.53
Fat-free mass, kg	DHEA Placebo		63.0±1.2 62.1±1.2	62.9±1.2 62.1±1.1	-0.1±0.3	0.81			43.3±1.1 44.2±1.2	43.9±1.2* 43.9±1.2	0.8±0.3	0.01
Trunk fat mass, kg	DHEA Placebo		14.5±1.0 11.9±0.9	14.1±1.0 12.3±0.9*	-0.9±0.3	0.009			14.1±0.9 14.7±0.9	14.0±0.9 14.8±1.0	-0.2±0.3	0.51
Appendicular fat mass, kg	DHEA Placebo		9.7±0.6 8.8±0.6	9.2±0.6* 9.0±0.6	-0.7±0.2	0.0001			14.2±1.0 15.3±1.1	14.1±1.0 15.4±1.1	-0.2±0.3	0.52
VAT, cm3	DHEA Placebo		2151±167 1720±218	2065±168* 1765±214	-117±62	0.06			932±106 906±92	909±108 895±90	-11±44	0.81
SAT, cm3	DHEA Placebo		2021±139 1694±102	1970±134 1773±97	-96±61	0.12			2177±187 2282±149	2194±191 2305±144	-9±61	0.88

* p≤0.05 versus baseline within group and gender. Visceral and subcutaneous adipose tissue volumes exclude data from 12 participants who did not undergo MRI scans due to metal prostheses or implants. VAT, abdominal visceral adipose tissue volume; SAT, abdominal subcutaneous adipose tissue volume.

During the second year of DHEA replacement, body weight, total fat mass and trunk and appendicular fat masses did not change significantly in the men (data not shown). The men who crossed-over from placebo to DHEA had no significant changes in body weight or body fat during the 12 mo of DHEA replacement. The only change in body composition in the women who crossed over from placebo to 12 mo of DHEA was a 0.5 ± 0.2 kg (P<0.01) increase in fat free mass.

### Oral Glucose Tolerance Test

**Figure 2.**
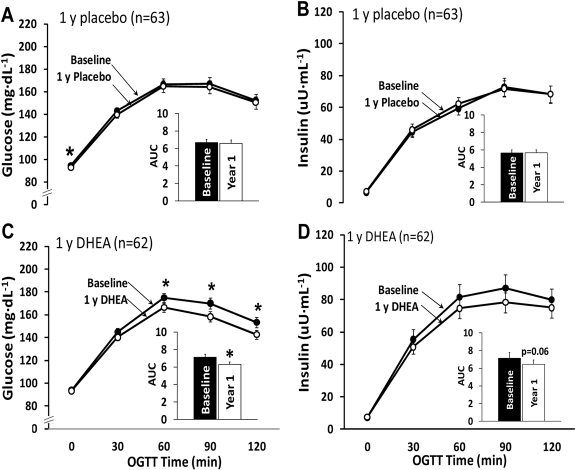
Oral glucose tolerance test results from the 1-yr randomized placebo-controlled trial. Changes in the areas under the curve for glucose and insulin did not differ significantly between the placebo group (panels A and B) and the DHEA group (panels C and D) (glucose, p=0.09; insulin, p=0.52). *p<0.05 for baseline to 1-yr change within group. AUC, area under the curve.

One year of DHEA replacement resulted in significant decreases in the glucose values at the 60 min, 90 min and 120 min time points of the oral glucose tolerance test (OGTT) and in the glucose area under the curve (AUC) (Figure 2C). A similar effect of DHEA replacement was seen in the participants in the placebo group who crossed over to DHEA in the second year open label study (data not shown). Insulin levels during the OGTT tended to be lower after one year of DHEA replacement, but the decrease did not attain statistical significance (p=0.06 for change in area under the insulin curve) (Figure 2D). However, the product of the glucose AUC and insulin AUC, an indicator of insulin resistance [11], was significantly improved, i.e. reduced, in response to one year of DHEA replacement (~22%, p,0.01), and was unchanged in the placebo group.

**Figure 3.**
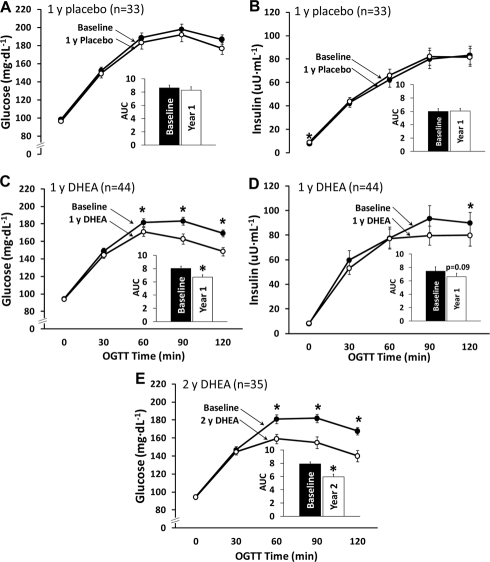
Oral glucose tolerance test results for subjects with abnormal glucose tolerance at baseline. The reduction in the glucose area under the curve (AUC) in the DHEA group (panel C) was significantly greater than that for the placebo group (panel A, p=0.03). Changes in insulin area under the curve did not differ between groups (panels B and D, p=0.52). Improvements in the glucose area under the curve were maintained in a subset of DHEA group participants who underwent a second year of DHEA supplementation (panel E). *p<0.05 for baseline versus DHEA.

It was evident on evaluation of the data, that improvements in glucose tolerance in response to DHEA occurred only in those participants who had abnormal glucose tolerance. We, therefore, did a separate analysis on the glucose tolerance data of the participants who had abnormal glucose tolerance initially. The improvements in glucose tolerance and AUC after one year (Figure 3C) of DHEA replacement was considerably greater when the results are not diluted by the values obtained on the participants with normal glucose tolerance. There were no significant changes in insulin levels during the OGTT except for the 120 min value, which was lower after 12 mo of DHEA (Figure 3D). The improvements in glucose tolerance persisted during the additional 12 mo of open label DHEA replacement (Figure 3E). There was no improvement in glucose tolerance in the participants with normal glucose tolerance.

### Hormone Concentrations

The hormone data on most of the participants in this study have been reported previously in a paper describing the effects of the DHEA replacement on bone mineral density [12]. In both men and women, the DHEA replacement increased plasma DHEAS into the young normal range (men 68±7 to 371±23μg/dl; women 41±5 to 293±21 μg/dl). In the men, DHEA replacement resulted in small, but statistically significant increases in testosterone, estradiol and their free indexes (~12%, p<0.05). In the women, testosterone and estradiol levels, and their free indexes increased about 2-fold in response to DHEA replacement.

### Plasma Lipids

DHEA replacement resulted in a significant decrease in plasma triglycerides (DHEA: 120±10 mg/dl to 105±8 mg/dl; Placebo: 103±8 mg/dl to 110±8 mg/dl; p<0.05 within DHEA group; p<0.05 between groups). There were no significant effects of DHEA on total, LDL, or HDL cholesterol in the men. DHEA replacement resulted in a 9% decrease in HDL cholesterol in women (p<0.01). This decrease had reversed by the end of the second year of DHEA replacement (initial 58±2, 12 mo 53±2, 24 mo 56 ± 2 mg/dl, means ± SE).

### Inflammatory Cytokines

There were significant decreases in the plasma concentrations of TNFα and IL-6 in response to 1 year of DHEA replacement (Table 3).

**Table 3.** Circulating Inflammatory Cytokines

	DHEA Group	Placebo Group	Adjusted Difference Between Groups	Between Group P value
TNF-α, pg/mL Baseline 12 months Change Within group P value	1.43 ± 0.13 1.25 ± 0.09 -0.18 ± 0.09 0.18	1.34 ± 0.16 1.62 ± 0.22 0.27 ± 0.16 0.04	-0.43±0.18	0.02
IL-6, pg/mL Baseline 12 months Change Within group P value	2.73 ± 0.20 2.32 ± 0.15 -0.41 ± 0.13 0.004	2.45 ± 0.16 2.93 ± 0.17 0.48 ± 0.15 0.0008	-0.78±0.17	<0.0001
Abbreviations: TNFα, tumor necrosis factor α; IL-6 interleukine-6

### Adverse Events

There were 12 serious adverse events and 124 minor events, with no difference in frequency between groups.

There were no serious adverse events attributable to DHEA replacement. Mild adverse events were development of acne in two women, which cleared up spontaneously, and increased facial hair growth in another woman. PSA levels averaged 1.10 ± 0.13 mg/ml at baseline and 1.07 ± 0.12 mg/dl after 12 mo in the men on DHEA, and 0.95 ± 0.13 mg/dl at baseline and 0.98 ± 0.12 mg/dl after 12 mo in the men in the placebo group. Based on mammograms and pap smears, no evidence of breast cancer or cervical abnormalities were detected in the women. Serum markers of liver function were unaffected by DHEA replacement.

## DISCUSSION

In our previous study, 6 mo of DHEA replacement resulted in an improvement in insulin action evidenced by a smaller increase in insulin during an OGTT without a change in glucose response [10]. In the present study, a longer period of DHEA replacement resulted not only in a reduction in insulin resistance, but also in improved glucose tolerance, in elderly, overweight or obese women and men with abnormal glucose tolerance. The magnitude of this improvement was impressive, as DHEA replacement for 1 yr resulted in an 18 mg/dl decrease in the 2h glucose value of the OGTT (p,0.02 vs. placebo) and 2 yr of DHEA resulted in a 21 mg/dl in the 2h glucose value. These findings have relevance to the current epidemic of impaired glucose tolerance and type 2 diabetes [13] because they raise the possibility that DHEA could be an effective treatment for impaired glucose tolerance and for prevention of type 2 diabetes.

Visceral fat, measured by MRI, decreased ~10% in both men and women [10]. This finding, together with the observation that DHEA protects laboratory rodents against visceral fat accumulation and insulin resistance with aging [7] and in response to high fat diets [5,6], led us to conclude that DHEA improves insulin action largely by reducing intra-abdominal fat [10].

While the present results confirm that DHEA replacement decreases insulin resistance in individuals with abnormal glucose tolerance, they do not support our previous conclusion that this improvement is largely mediated by a decrease in visceral fat. In the present study, the women had a small increase in fat free mass without a decrease in abdominal fat, while the men had only a small decrease in visceral fat in response to DHEA, suggesting that the reduction in visceral fat observed in our previous, 6 mo study is a transient effect.

In addition to our earlier study [10], other studies have found an improvement in insulin action in response to DHEA replacement, including in hypoadrenal women [13], middle-aged hypercholesterolemic men [14], and postmenopausal women [15]. However, in contrast to the present results, it was recently reported by Nair et al. [16] and Basu et al. [17] that two years of DHEA treatment had no effect on insulin action or postprandial glucose turnover in elderly men and women. The difference between the results of their study and ours, despite a similar study design, would be puzzling were it not for our finding that DHEA replacement improved glucose tolerance/insulin resistance only in those participants who had abnormal glucose tolerance, with no improvement in those with normal glucose tolerance. Although Nair et al. [16] and Basu, et al. [17] did not perform a standard OGTT, it appears that their subjects were unusually insulin sensitive for their age. This is evidenced by a fasting insulin level of ~3.6 μU/ml, a value similar to that of their young controls and about 50% lower than that of our participants with abnormal glucose tolerance, a fasting glucose value ~5 mgdl lower than that of our participants, and a calculated HOMA index [18] of insulin resistance of 0.88 compared to 1.88 for our participants with abnormal glucose tolerance. Thus, the lack of effect of DHEA on insulin action in the study by Nair et al. [16] and Basu et al. [17] is in keeping with our finding that DHEA improves insulin action only in individuals with abnormal glucose tolerance.

Regarding mechanisms by which DHEA may reverse insulin resistance, one possibility is by activation of PPARα for which DHEA is a ligand [19,20]. Activated PPARα stimulates expression of the mitochondrial enzymes involved in fat oxidation and represses activation of enzymes involved in fat synthesis[20,21]. As a result, PPARα activation lowers triglycerides [21], and could result in less fat deposition in liver and muscle. PPARα activation also suppresses inflamemation [21], and DHEA has been shown to inhibit nuclear factor Kappa B and production of IL-6 and TNFα by various cell types, and to lower circulating levels of these inflammatory cytokines [22-26]. Chronic inflammation in adipose tissue and macrophages is thought to be one of the factors that mediates insulin resistance [27,28]. In the present study, DHEA replacement resulted in significant reductions in plasma IL-6 and TNFα, suggesting a decrease in inflammation. It is of interest relative to a role of PPARα activation in the improvement in insulin action induced by DHEA, that fibrates, which mediate their effects on lipid metabolism by activating PPARα [21], have also been reported to improve insulin action and glucose tolerance [29-36].

Another mechanism by which DHEA or some of its metabolites might enhance insulin action is by improving impaired activation of phosphatidyl inositol 3-kinase and Akt/PkB by insulin in insulin resistant muscle [37-39]. It is interesting in this regard that 17α-ethynyl-5-androstene-3β, 7β, 17β triol, an analog of a DHEA metabolite, enhances insulin-stimulated Akt/PkB phosphorylation in muscle, and improves muscle and liver insulin sensitivity and glucose tolerance in Zucker diabetic rats and improves insulin sensitivity in insulin resistant humans [37].

In conclusion, the results of this study confirm our previous finding that DHEA replacement in elderly men and women improves insulin action [10]. They further show that a longer period of DHEA treatment than was evaluated in our previous study, 12 mo vs. 6 mo, also significantly improves glucose tolerance, and that this improvement occurs only in those individuals who have abnormal glucose tolerance. These findings raise the possibility that DHEA could become a first line treatment for glucose intolerance/insulin resistance and diabetes prevention in older individuals and ameliorate the increase in chronic inflammation that occurs with aging.

## METHODS

### Participants

Men and women, aged 65-75y were recruited from the Saint Louis area. Screening included a medical history, physical examination, blood chemistry analysis, hematology, urinalysis, and electrocardiography. Volunteers were excluded if they were current smokers or if they had evidence of chronic infection, a history or evidence of malignancy within the past 5 yr, clinical cardiovascular disease, advanced emphysema, advanced Parkinson's disease, resting blood pressure >170 mmHg systolic or >100 mmHg diastolic, or diabetes (self-report or fasting plasma glucose ≥ 126 mg/dL). Participants taking medications were required to maintain stable dosing regimens for six months prior to the study. Informed written consent was obtained from the participants, and the study was approved by the Human Research Protection Office at Washington University School of Medicine.

### Intervention

Participants were randomized to 12 months of 50 mg/d DHEA or placebo. Both groups received multivitamin and calcium/vitamin D supplements and were advised to maintain their usual diet and physical activity. During monthly meetings with the study nurse, participants received a month's supply of DHEA or placebo, had pill counts performed, and were questioned about adverse events, changes in activity level, diet, and medications.

### Oral Glucose Tolerance Test (OGTT)

OGTTs (75g) were started in the morning after an overnight fast. The study dietitian instructed the participants to consume ≥ 250 g/day carbohydrate for the three days preceding the test. Plasma glucose and insulin were measured using the glucose oxidase method (model 2300 Stat Plus, YSI Inc., Yellow Springs, Ohio) and radioimmunoassay [40]. Incremental area under the curve (AUC) was calculated for OGTT glucose and insulin values by the trapezoidal method [41]. Because of technical problems year 2 insulin data were not available. Although volunteers with self-reported diabetes or a fasting plasma glucose ≥126 mg/dL were excluded from the study, results from the baseline OGTT indicated that 16 of the enrolled participants had diabetes based on a 2-hr glucose ≥200 mg/dL. These participants were not excluded.

### Fasting Blood Analyses

Plasma total cholesterol, HDL-cholesterol, and glycerol-blanked triglycerides (TGs) were measured by a CLIA-certified clinical laboratory using automated enzymatic/colorimetric assays (Roche/Hitachi Modular Analytics System; Roche Diagnostics Corporation, Indianapolis, IN). Tumor necrosis factor-α (TNFα) and interleukin-6 (IL-6) (Quantikine High Sensitive, R&D Systems, Minneapolis, MN) were measured by using ELISA.

### Body Composition, Body Mass, and Height

Whole body fat mass and fat free mass, Truncal fat and appendicular fat mass, were measured by dual X-ray absorptiometry (DXA) (Delphi W, software version 11.2; Hologic Corporation, Waltham MA). Visceral and subcutaneous abdominal adipose tissue volumes were measured using magnetic resonance imaging. Ten serial 10-mm axial images were acquired using a 1.5-T super-conducting magnet (Siemens, Iselin, NJ), beginning at the first lumbar vertebra and moving downward. Images were batch analyzed with HIPPO software (version 1.3, Pisa, Italy) [42,43]. Slice level fat volumes were summed to get total adipose tissue volumes for the region of interest.

### Physical Activity

Physical activity levels were assessed using the Aerobic Center Longitudinal Study Physical Activity Questionnaire [44]. The questionnaire is designed to assess habitual physical activity performed over the past 3 months.
